# Estimation of a Killer Whale (*Orcinus orca*) Population’s Diet Using Sequencing Analysis of DNA from Feces

**DOI:** 10.1371/journal.pone.0144956

**Published:** 2016-01-06

**Authors:** Michael J. Ford, Jennifer Hempelmann, M. Bradley Hanson, Katherine L. Ayres, Robin W. Baird, Candice K. Emmons, Jessica I. Lundin, Gregory S. Schorr, Samuel K. Wasser, Linda K. Park

**Affiliations:** 1 Conservation Biology Division, Northwest Fisheries Science Center, National Marine Fisheries Service, National Oceanic and Atmospheric Administration, 2725 Montlake Blvd E, Seattle, Washington, 98112, United States of America; 2 Center for Conservation Biology, Department of Biology, University of Washington, Seattle, Washington, 98195, United States of America; 3 Cascadia Research Collective, Olympia, Washington, 98501, United States of America; Sonoma State University, UNITED STATES

## Abstract

Estimating diet composition is important for understanding interactions between predators and prey and thus illuminating ecosystem function. The diet of many species, however, is difficult to observe directly. Genetic analysis of fecal material collected in the field is therefore a useful tool for gaining insight into wild animal diets. In this study, we used high-throughput DNA sequencing to quantitatively estimate the diet composition of an endangered population of wild killer whales (*Orcinus orca*) in their summer range in the Salish Sea. We combined 175 fecal samples collected between May and September from five years between 2006 and 2011 into 13 sample groups. Two known DNA composition control groups were also created. Each group was sequenced at a ~330bp segment of the 16s gene in the mitochondrial genome using an Illumina MiSeq sequencing system. After several quality controls steps, 4,987,107 individual sequences were aligned to a custom sequence database containing 19 potential fish prey species and the most likely species of each fecal-derived sequence was determined. Based on these alignments, salmonids made up >98.6% of the total sequences and thus of the inferred diet. Of the six salmonid species, Chinook salmon made up 79.5% of the sequences, followed by coho salmon (15%). Over all years, a clear pattern emerged with Chinook salmon dominating the estimated diet early in the summer, and coho salmon contributing an average of >40% of the diet in late summer. Sockeye salmon appeared to be occasionally important, at >18% in some sample groups. Non-salmonids were rarely observed. Our results are consistent with earlier results based on surface prey remains, and confirm the importance of Chinook salmon in this population’s summer diet.

## Introduction

Correctly understanding relationships between predators and prey, including an accurate understanding of predator diets, is often important for well-informed management of both endangered predators and prey. Recent examples include concerns about adequate levels of prey for threatened predators (e.g., Steller sea lions [1]), predation on domesticated populations by reintroduced predators (e.g., grey wolves [2, 3]), predation on threatened fish species by abundant pinniped or bird populations [4, 5], and competition between rare and abundant predators for a common prey source [6]. In response to conservation concerns related to predation, fish and wildlife managers have at times employed a variety of potentially expensive and disruptive actions, such as fisheries closures, and lethal predator removal [7].

Concerns about an endangered killer whale (*Orcinus orca*) population in the eastern North Pacific provide a good example of a complex conservation problem involving predators and their prey. As a species, killer whales have a global distribution and a diverse diet [8]. However, the species is subdivided into numerous discrete populations, many of which specialize on specific prey types [8]. Such specialization has been most thoroughly documented in the nearshore temperate North Pacific Ocean, where two genetically discrete sympatric killer whale ‘ecotypes’ feed on either marine mammals or fish, respectively [9, 10]. The fish-eating killer whales (for historical reasons often referred to as ‘residents’) and mammal-eating killer whales (also known as Bigg’s killer whales or ‘transients’) differ at a sufficient number of traits that some investigators have suggested they are incipient species [11–13]. As a result of such prey specialization, individual killer whale populations may be particularly vulnerable to declines of their favored prey.

Low prey abundance has been cited as an important factor limiting the recovery of an endangered population of fish-eating killer whales, known as the southern resident population [14]. This population, which consists of three socially defined pods (J, K and L) [15], is the southern-most distributed of the fish-eating populations inhabiting the northeast Pacific Ocean, and ranges in coastal waters from central California to northern British Columbia [16]. Previous studies based on morphological and genetic analysis of prey remains found near the water surface following kills have indicated that Pacific salmon (*Oncorhynchus spp*.), and in particular Chinook salmon, *O*. *tshawytscha*, make up >90% of this population’s summer diet [9, 10, 17]. Many stocks of Chinook salmon are also listed as threatened or endangered [18], and lack of this preferred prey item has been associated with poor survival and fecundity of the southern resident killer whales [19, 20].

Analysis of prey remains found near the surface after predation events has proven useful for diet estimation, but may be potentially biased if some prey types are more likely than others to be consumed near the surface or broken up before consumption [9, 17]. Analysis of DNA isolated from feces is a potentially useful additional source of diet information, both because it may be less biased toward items consumed near the surface and because each fecal sample may integrate information over multiple feeding events. A previous study of killer whale diet that used PCR methods to determine if killer whale feces contained DNA from specific prey items in fact did identify more taxa in the feces than were present in samples of prey remains, but the method did not allow for the quantification of relative DNA amounts [17].

An accurate understanding of the diet of the southern resident killer whale population is of importance to management decisions impacting both the whales and their prey [21]. Molecular analysis has long been used to study species presence in feces [22–24]. More recently, quantitative analysis of DNA sequences isolated from fecal samples using high-throughput sequencing has been shown to be a useful tool for semi-quantitative diet estimation (reviewed by [25]). Such analyses may be particularly useful for species, such as cetaceans, for which predation events are difficult to observe directly (e.g., [26]). Here, we report on a study using high-throughput sequencing of DNA from fecal samples to estimate the diet composition of the southern resident killer whale population in its core summer range.

## Methods

### Field Methods and Sampling

**Fig 1:**
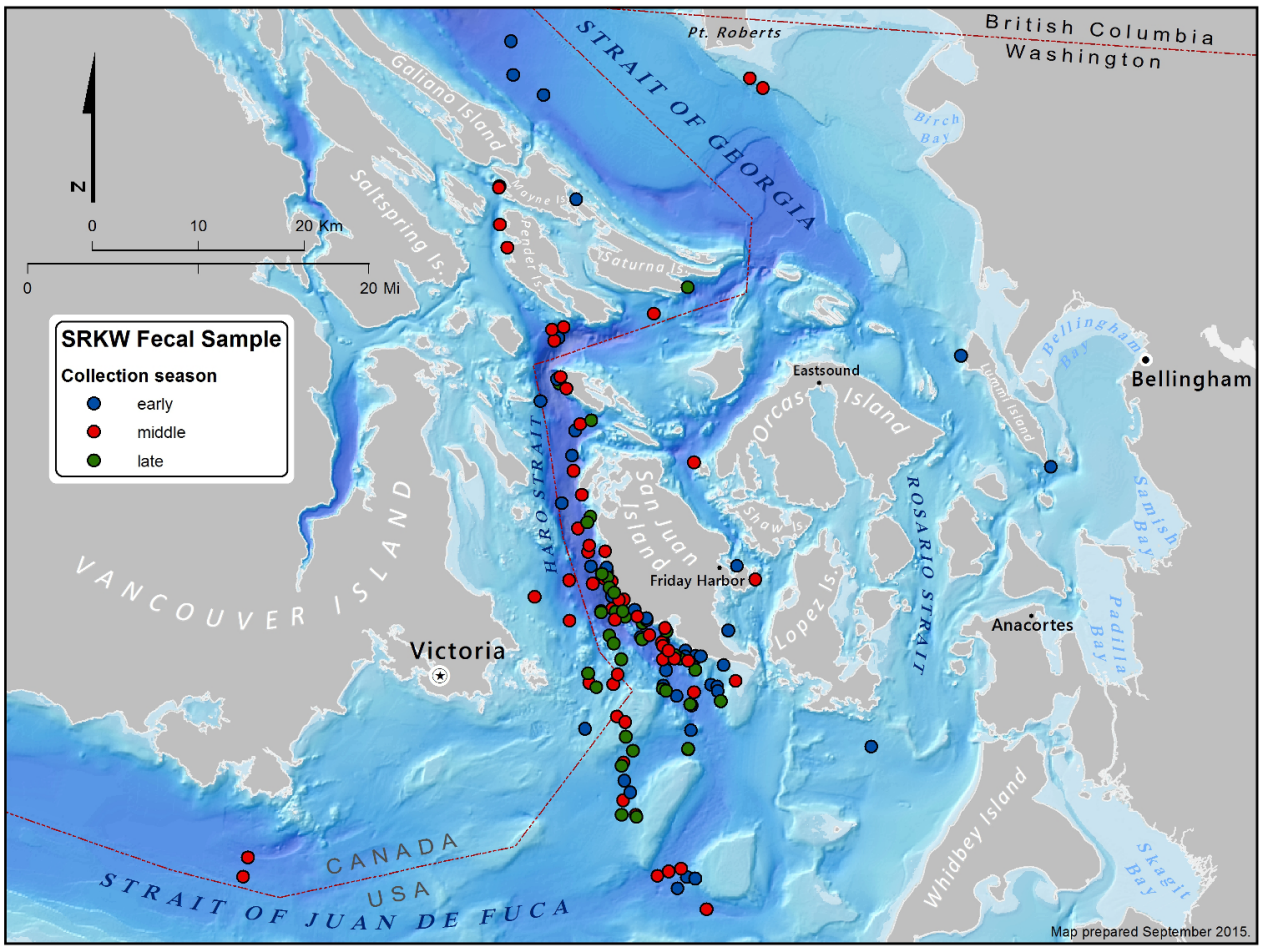
Sampling locations of fecal material. Each dot represents one sampling event, color coded to represent sampling occurring in early (May-July 25), mid (July 26-September 4), and late (September 5–30) summer.

Field activities were based out of the San Juan Islands between May and September of 2006–2011 (Fig 1). Fecal samples were collected using two different previously described techniques: 1) a modification of a method developed by Ford and Ellis [9] for prey sampling that involved following a focal animal’s ‘‘fluke prints” until a sample was observed (for additional details, see [17]) or 2) using scent detection dogs to locate samples floating on the water’s surface [27]. Samples were initially stored in plastic bags on ice packs and later stored at -20 C or -80 C prior to analyses. Samples were collected in U.S. waters under NMFS General Authorization No. 781–1725, and NMFS Scientific Research Permits 781-1824-01, 16163, 532-1822-00, 532–1822 and 10045. Samples were collected in Canadian waters under Marine Mammal License numbers 2008–16, 2009–08, 2010–09 and 2012–08, and Species at Risk Act permits numbered 91, 102, 109 and 155. Sample collection methods were approved by the University of Washington’s Institutional Animal Care and Use Committee under protocol 2850–08.

**Fig 2:**
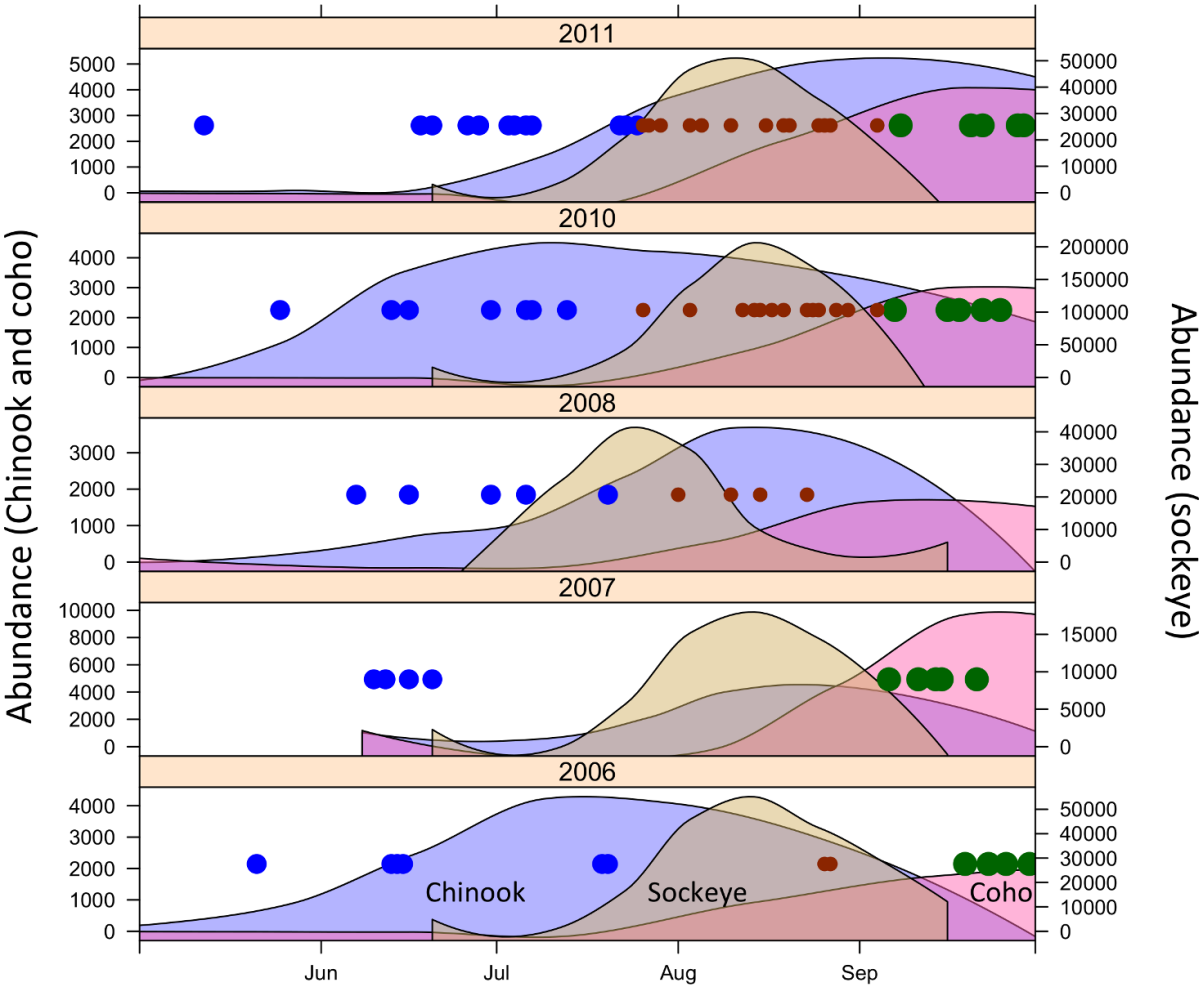
Temporal distribution of fecal samples included in the analysis and of the approximated daily abundance of Chinook, sockeye and coho salmon in the San Juan Islands area. Each dot represents fecal samples collected on a specific day, with the area of the dot proportional to the number of samples on that day (smallest size = 1 sample). The dots are color-coded to indicate the within-year pools of samples that were combined for sequencing analysis, for a total of 13 year-by-season sample pools. Smoothed daily salmon abundance was estimated by local polynomial regression of daily catch-per-unit-effort data scaled by total annual run size (see S1 Text for details).

An initial group of 244 fecal samples (subsequently reduced to 175 based on DNA quality considerations—see below) were sorted by sample collection date into 13 experimental groups (Fig 2; S1 Table). The groupings were designed to allow for comparisons between years and to capture shifts in the proportions of prey species consumed throughout the course of the late spring and summer. Samples were divided by year and samples collected in the same year were subdivided into groups using the following date ranges: early summer (May–July 25^th^), mid-summer (July 26^th^–September 4^th^), and late summer (September 5^th^– 30^th^) (Fig 2). The number of individual fecal samples contributing to each group varied due to differences in sampling effort and whale presence during different years and seasons. The date ranges for the groupings were selected after sample collection, considering natural breaks in the temporal sequence of samples and the number of samples in each grouping. The largest group included 32 samples and the smallest group included 4 samples (Fig 2; S1 Table).

### DNA Extraction

We extracted DNA from fecal samples using a Qiagen QiaCube and the QIAamp DNA Stool Mini Kit (Qiagen, Valencia, CA) in a polymerase chain reaction (PCR)-free laboratory. Fecal samples affixed to substrate (sterile gauze or swabs) were trimmed and transferred into a 2mL extraction vial and feces were lysed directly on the substrate. For fecal samples not affixed to a substrate, DNA was extracted from approximately 200uL of feces. Samples containing large quantities of seawater were centrifuged at low speed prior to extraction to pellet the feces and excess seawater was decanted. For all fecal samples, DNA was eluted off extraction columns by incubating 100uL of elution buffer for 5 minutes at room temperature prior to centrifugation.

To confirm that each fecal sample was indeed killer whale feces, we amplified and sequenced the 16s region of the mitochondrial genome [28] and compared the sequence with known killer whale sequence. Samples were only collected in the proximity of known southern resident killer whale groups, and thus were unlikely to be from other populations of killer whales. In addition, the fecal samples were also genotyped at a series of microsatellite loci as previously described [27, 29] to confirm the presence of killer whale DNA and in many cases identify the specific pod or individual of origin (S1 Table).

### Prey Detection Primer Design

Previous work [9, 10] has shown that the southern resident killer whale population does not prey on marine mammals, so our study focused exclusively on detecting fish prey. PCR primers were designed to amplify approximately 330bp of the 16s region of the mitochondrial genome of a wide range of potential fish prey species while excluding amplification of killer whale DNA. Primers were selected based on an alignment of more than 40 potential prey species representing all major fish families (S2 Table). Species were chosen based on previous studies or common presence in the whales’ habitat. To avoid amplification bias among taxa, PCR was performed using an equimolar mix of two forward primers (Table 1). Primer “Salmon-F” is complementary to the priming site in salmonids and several non-salmonids and “Groundfish-F” is complementary to the sequence in most non-salmonids (S2 Table). PCR product length (excluding primers) ranged from 326bp to 341bp depending on the species.

**Table 1:** PCR primer sequences.

Primer	Sequence 5' to 3'*
Salmon-F	**TCGTCGGCAGCGTC**AGATGTGTATAAGAGACAGg**c**aatcacttgtcttttaaatgaagacc
Groundfish-F	**TCGTCGGCAGCGTC**AGATGTGTATAAGAGACAGg**t**aatcacttgtcttttaaatgaagacc
16s-R	**GTCTCGTGGGCTCGG**AGATGTGTATAAGAGACAGggattgcgctgttatcccta

*Overhang adaptor sequence in uppercase bold, locus-specific sequence in lowercase.

Primers were tested on the following known species: Chinook salmon (*Oncorhynchus tshawytscha*), coho salmon (*O*. *kisutch*), chum salmon (*O*.*keta*), steelhead (*O*. *mykiss*), sockeye salmon (*O*.*nerka*), lingcod (*Ophiodon elongates*), Pacific halibut (*Hippoglossus stenolepis*), English sole (*Parophrys vetulus*), Pacific herring (*Clupea pallasii*), and killer whale (*Orcinus orca*). PCR products were visualized by gel electrophoresis and showed that DNA from all fish species amplified successfully, while the killer whale DNA produced no discernible PCR product.

The prey detection primers were subsequently modified to allow for indexing of individual pools during the library preparation step. Both forward primers (Salmon-F and Flatfish-F) and the reverse 16s-R were modified by the addition of Illumina primer sequence and overhang adaptors complementary to Illumina’s Nextera index tag kit on the 5’ ends (Table 1). Primers were HPLC purified to remove truncated primers that might compromise amplification specificity.

### Quality Assessment and Sample Pooling

In order to assess the quality and initial quantity of prey DNA within each fecal sample, all fecal DNA extractions were screened via qPCR. The qPCR assay was designed to amplify a smaller (approximately 87bp) sequence nested within the 330bp 16s region described above. The assay used the same forward primers as the prey detection assay in combination with the reverse primer 16s-short-R: 5’- tccatagggtcttctcgtctt. Each reaction was carried out in a final volume of 12.5uL using 6.25uL of Power Sybr Green Master Mix (Applied Biosystems), 0.2uM of forward primers, 0.2uM of reverse primer, and 2uL of DNA. Reactions conditions were as follows: 95°C for 10min, followed by 40 cycles of 95°C for 15 sec; 60°C for 1 min, followed by a melt curve analysis. All reactions were performed using the ABI 7900HT Fast Real-Time PCR System. Each qPCR assay plate included a 1:10 dilution series of prey DNA standard. The prey DNA standard was created by pooling normalized DNA (quantified fluorometrically via a Qubit) from 8 fish species (the six salmon species plus halibut and English sole–Table 2). Fecal DNA extractions that failed to amplify or amplified poorly using the qPCR assay were removed from further analysis. A total of 175 samples remained after qPCR evaluation. Individual fecal DNA extractions were quantified against the standard curve, normalized within a group (as identified above), and pooled into one of 13 discrete pools of fecal DNA (see above for pool definitions). The normalized DNA pools were re-screened using qPCR to verify normalization.

**Table 2:** Reference Sequences used for species assignment.

Common Name	Scientific Name	Length (bp)	GenBank Reference
Chinook salmon	*Oncorhynchus tshawytscha*	326	KU170128-31
Coho salmon	*Oncorhynchus kisutch*	326	KU170132-34
Steelhead	*Oncorhynchus mykiss*	326	KU170139-40
Chum salmon	*Oncorhynchus keta*	325	KU170137-38
Sockeye salmon	*Oncorhynchus nerka*	325	KU170135-36
Pink salmon	*Oncorhynchus gorbuscha*	325	EF455489.1
Spiny dogfish	*Squalus acanthias*	326	EF119335.1
Dover sole	*Microstomus pacificus*	326	FJ870397.1
English sole	*Parophrys vetulus*	326	EF119289
Pacific hake	*Merluccius productus*	326	EF458338.1
Pacific halibut	*Hippoglossus stenolepis*	326	FJ870421.1
Pacific herring	*Clupea pallasii*	326	EF458434.1
Lingcod	*Ophiodon elongates*	326	EF458353.1
Killer whale	*Orcinus Orca*	326	EU685093.1
Spotted ratfish	*Hydrolagus colliei*	326	EF119279.2
Quillback rockfish	*Sebastes maliger*	326	EF446599.1
Sablefish	*Anoplopoma fimbria*	326	EF458482.1
Surf smelt	*Hypomesus pretiosus*	326	EF458416.1
Shiner perch	*Cymatogaster aggregata*	326	EF119256.1

In addition to the experimental pools, two control pools were created by combining DNA extracted from 5 known southern resident killer whale fish prey species: Chinook, coho, and sockeye salmon; halibut and English sole. To create the control pools, genomic DNA was extracted from finclips or fish muscle tissue and screened via gel electrophoresis. High quality, high molecular weight genomic DNA samples were selected and quantified with the qPCR assay used to normalize fecal DNA extractions. The first control pool consisted of equal proportions of Chinook, sockeye, halibut and English sole DNA. The second control pool was made up of 40% halibut, 40% sockeye, 15% coho and 5% Chinook DNA.

### Amplicon Library Preparation and Sequencing

Amplicon libraries were generated from each of the 13 experimental pools and 2 control pools using a 2-step PCR workflow provided by Illumina. The first PCR employed the modified prey detection primers to generate template libraries of individual pools: 13 separate 40uL reactions, each containing 4uL of one DNA pool, 1X Promega GoTaq Flexi buffer (Promega Corp., Madison WI), 3.0mM MgCl_2_, 0.2mM of each dNTP, 0.1ug/uL of BSA, 0.2uM of each primer, and 2 units of Promega GoTaq Flexi DNA Polymerase. PCR cycling conditions included an initial denaturation at 94°C for 2 min, followed by 32 cycles of 94°C for 35 sec; 61°C for 1 min; 72°C for 35 sec; and a final extension at 72°C for 5 min. PCR-setup was performed in a PCR-product free laboratory using aerosol-resistant pipette tips. Amplicon libraries were gel purified to remove unspecific PCR products, primers, unincorporated nucleotides, dyes and polymerase.

The second step of the workflow was a limited-cycle PCR that used the overhang adaptors to append P5 and P7 Illumina sequencing adapters and Illumina Nextera XT index tags to amplicons generated during the first round of PCR. The second PCR step was performed in 50uL reactions containing 8uL of gel purified PCR product from the first-step PCR, 1X NEB Phusion HF DNA Polymerase buffer (Ipswich, MA), 0.2mM of each dNTP, 5uL each of one Illumina Nextera forward and reverse index tag, and 1 unit of NEB Phusion HF DNA Polymerase. Each of the 15 DNA pools amplified in PCR #1 were re-amplified with a unique combination of 1 of 6 Nextera forward primers containing barcodes N01-N06 and 1 of 4 Nextera reverse primers containing barcodes S501-S504. We used PCR cycling conditions specified by the Illumina Nextera amplicon sequencing protocol as follows: 72°C for 3 min, 98°C for 30 sec, followed by 12 cycles of 98°C for 10 sec, 55°C for 30 sec, 72°C for 30 sec, and a final extension at 72°C for 5 min. The 15 indexed amplicon libraries were gel purified, quantified using the KAPA SybrGreen Library Quantification Kit (KAPA Biosystems, Boston, MA), normalized, and pooled prior to sequencing. The final pool of libraries was normalized to 10nM. In preparation for sequencing on an Illumina MiSeq, the pool was further diluted to 10pM and 5% PhiX was added to create diversity, optimize cluster formation on the MiSeq flowcell, and allow for real-time quality metrics during the run. Finally, 250bp paired-end sequencing was performed using a MiSeq Reagent Kit v2 for 500 cycles.

### Data Analysis

Using the Illumina MiSeq Reporter Software, the indexed reads were de-multiplexed and FASTQ files were generated for each library. Reads were trimmed to remove adaptor and index sequences prior to further analysis. Because the length of the amplicon was ~330bp, there was a 65-100bp region of overlap between the paired reads. We used the PANDAseq [30] software program to align and merge the paired reads into a single sequence, using the default alignment threshold of 0.6.

Merged reads were aligned against a reference sequence database using the BLAST+ command line program [31]. The reference sequence database consisted of the target 16s amplicon from 19 species including 6 salmonids (Chinook, coho, steelhead, sockeye, chum and pink salmon); 3 species of flatfish (Pacific halibut, Dover sole, and English sole); as well as lingcod, sablefish, rockfish, hake, herring, smelt, surfperch, dogfish, ratfish and killer whale (Table 2). To account for intraspecific variation among the relatively closely related salmon species, we included 2–4 haplotypes for each salmon species except for pink salmon (Table 3). Most species pairs differed by >50bp, although some species, including the six salmon species, differed by <10bp (S3 Table). In order to further validate the power of the baseline to distinguish among the six Pacific salmon species, we also searched GenBank for 16s *Oncorhynchus* sequences, and found 40 such sequences (S4 Table). Of these, 39 assigned to the ‘correct’ species when using our baseline, the one exception being a putative *O*. *mykiss* sequence that was identified as *O*. *kisutch* using BLAST (S4 Table). This sequence, however, appears to have obtained from an unlabeled specimen in a Hong Kong market, and may therefore have been mislabeled [32]. Overall, however, this segment of the 16s gene appears to have sufficient power to distinguish among species of interest. Species identifications of the unknown sequences were based on the closest match to the reference database, after first removing alignments of <320 bp, >1 bp gap, and <95% sequence similarity to the best match in the database to reduce spurious assignments due less than full length or poor quality sequences. Sequences that had identical alignment scores to two different species were removed from further analysis.

**Table 3:** Expected and observed proportions of sequences in the experimental controls.

	Control 1		Control 2	
Species	Exp.	Obs	Exp.	Obs.
Halibut	0.250	0.205	0.400	0.344
English sole	0.250	0.203	0.000	0.000
Chinook	0.250	0.307	0.050	0.120
Sockeye	0.250	0.284	0.400	0.387
Coho	0.000	0.000	0.150	0.149

## Results

A total of 13,769,809 sequence reads were generated of which 12,586,467 (91.4%) passed the initial filter specified by the Illumina MiSeq software. Of the reads that passed the filter, 79.3% were assigned to an index. After assembling the paired reads, there were 5,168,233 total sequences, ranging from 228,906 to 518,974 sequences per group. Over the 13 pools, the mean sequence identity and length of the best-fit alignment for each of the sequences were 99.25% and 324.5 bp, respectively. After filtering for sequence length, gaps, and percent sequence identify (see above), 4,987,107 alignments remained with mean sequence identity and length of 99.5% and 325.8 bp, respectively. A total of 45,881 killer whale sequences were detected (0.9% of sequences), indicating that our primer design was generally successful at limiting amplification of host (killer whale) vs. prey DNA. For the two control pools, there were 667,983 paired reads, which were reduced to 591,593 after filtering for length, gaps, and percent sequence identity. A small number (<0.5%) of sequences resulted in identical blast scores to two different species, and these were removed from further analysis.

For the salmon sequences in the control pools, the BLAST reference sequences and the query sequences were derived from the same individuals, allowing for a rough characterization of sequencing error rates. The average percent sequence divergence between the aligned salmon control and query sequences was 0.6%, consistent with the 0.5–1.0% substitution error rate that has been previously reported for MiSeq sequencing [33].

The estimated species composition in the two control groups differed somewhat from expectations (Table 3). In control group 1 (equal proportions of four species), the two salmon species (Chinook and coho) were overrepresented (25% expected, 30% observed) at the expense of the two groundfish species (25% expected, 20% observed). In control group 2, sockeye and coho salmon were within 1 percentage point of their expected values (40% and 15%, respectively), and Chinook was overrepresented (5% expected, 12% observed) at the expense of halibut (40% expected, 34% observed).

Salmonids made up >98.6% of the sequences, with halibut and herring being the most abundant non-salmonids at <1% each (Table 4). Within sample groups, the lowest percentage of salmonid sequences was 90.5% in the mid-summer 2011 sample, with the remainder being primarily herring (9.4%). The late summer 2007 sample also had a relatively high percentage of non-salmonids (6.4% halibut). All other samples contained >99% salmonid sequences.

**Table 4:** Proportion of DNA sequences from potential prey species sequenced from killer whales fecal samples*.

Season	Year	Halibut	Herring	Chinook	Chum	Coho	Sockeye	Steelhead	Total salmon
Early	2006	0.000	0.000	0.981	0.009	0.005	0.000	0.000	0.995
Early	2007	0.000	0.000	0.990	0.003	0.003	0.000	0.002	0.999
Early	2008	0.000	0.000	0.961	0.001	0.000	0.020	0.016	0.999
Early	2010	0.000	0.000	0.999	0.001	0.000	0.000	0.000	1.000
Early	2011	0.000	0.000	0.969	0.001	0.001	0.000	0.029	1.000
Middle	2006	0.003	0.000	0.995	0.001	0.001	0.000	0.000	0.997
Middle	2008	0.005	0.000	0.873	0.001	0.000	0.121	0.000	0.995
Middle	2010	0.005	0.000	0.971	0.003	0.020	0.000	0.000	0.994
Middle	2011	0.001	0.094	0.721	0.000	0.001	0.183	0.000	0.905
Late	2006	0.000	0.000	0.297	0.001	0.607	0.000	0.094	0.999
Late	2007	0.064	0.000	0.756	0.004	0.172	0.001	0.002	0.936
Late	2010	0.000	0.002	0.532	0.027	0.438	0.000	0.000	0.997
Late	2011	0.001	0.000	0.475	0.001	0.522	0.001	0.000	0.999
Total		0.006	0.008	0.795	0.004	0.150	0.025	0.013	0.986

* The following species were included in the analysis but had <0.01 representation among the DNA sequences in all sample groups: lingcod, sablefish, rockfish, English sole, surf smelt, hake, dogfish, ratfish, and pink salmon.

Of the six salmonid species, Chinook salmon was the most common at 79.5% of the overall sequences (Table 4). Coho salmon was also relatively common (15% of sequences overall), and three other species (chum, sockeye and steelhead) had small overall contributions of <3% each. There were some clear patterns over the course of the summer. The early summer samples were >96% Chinook salmon in all five years. The mid-summer samples were also mostly Chinook salmon, but in 2008 and 2011 also contained some sockeye salmon (12.1% and 18.3%, respectively). The late summer samples had the lowest proportions of Chinook (30–75%, average of 51.3%), and contained substantial fractions of coho salmon (18–60%, average 43.5%). The samples were obtained from > 54 individuals across all three pods (S1 Table, Table 5), so these results are likely to be representative of the population as a whole rather than individual level variation. Most of the samples were obtained from the west side of San Juan Island (Fig 1), consistent with the whales’ frequent use of this area [34].

**Table 5:** Summary of pod origins of fecal samples. Each year/season (E = early; M = middle; and L = late summer, respectively) combination was one sample pool, with the exception of mid-summer 2007 and late summer 2008 for which there were no samples in the study.

	2006			2007			2008			2010			2011		
pod	E	M	L	E	M	L	E	M	L	E	M	L	E	M	L
Unk	2	4	2	4	0	1	0	0	0	1	3	1	5	6	3
J	6	1	1	9	0	4	3	2	0	3	10	6	10	4	2
K	6	0	2	0	0	0	3	1	0	0	4	1	4	10	4
L	0	2	1	2	0	15	0	1	0	4	4	3	1	12	2

## Discussion

Using high-throughput sequencing of DNA extracted from killer whale feces collected in the field, our results confirm earlier studies [9, 10, 17] indicating that salmon, and especially Chinook salmon, are by far the dominant component of the southern resident killer whale diet during the summer months. The 79.5% overall estimated proportion of Chinook salmon sequences for the entire May–September time period is nearly the same as the 80% a prior study obtained based on analysis of surface prey remains [17]. The second largest estimated diet component, coho salmon, was also similar in both studies, with 15% in the current study and 9% based on prey remains. The marked similarity in estimated diet composition between the fecal analysis and the prey remains analysis confirms the utility of the surface collection techniques [9], and suggests that at least during the summer time period the prey consumed near the surface are unlikely to be a taxonomically biased sample of the whales’ diet.

Despite the overall prevalence of Chinook salmon, there was also some evidence of dietary shifts over the course of the summer. Most notable was a marked shift toward coho salmon in the late summer in all four years for which we had late summer samples (a shift that was also observed in prey remains [17]). The other apparent seasonal shift was a spike (up to 18%) in sockeye salmon in mid-summer in two of the four years. A smaller spike of sockeye in mid-summer was also apparent in an earlier prey remains study on this population, but this was based on very limited data (a total of only 4 sockeye prey remains; [17]).

In a broad sense, these diet shifts from Chinook salmon to other salmon species generally coincide with the run timing of the salmon species to the San Juan Islands area. In particular, sockeye salmon and coho salmon have a mid- and late-summer run timing distribution, respectively, and these are also the time periods in which these species appear in the killer whale diet (Fig 2). However, our results also clearly confirm previous observations [9] indicating that the whales are not consuming the salmon species in proportion to their abundance. In particular, during the peak of their run, sockeye salmon are often greater than 10-fold more abundant than Chinook salmon, but Chinook salmon were estimated to contribute >70% of the whales’ diet, even during the peak of sockeye runs. Even in 2010 when a particularly large run of sockeye returned to the Fraser River (Fig 2) during a time period well sampled by our fecal analysis, the estimated contribution of sockeye to the whales’ diet was close to zero (Table 4). Similarly, the Fraser River has a large run of pink salmon that also returns in mid-summer in odd-numbered years, but pink salmon sequences were entirely absent from the fecal samples. The primer regions for pink salmon are identical to the other species (S2 Table), so PCR failure is not a plausible explanation for the absence of this abundant species from the fecal samples.

In contrast, the shift toward coho salmon in the late summer clearly does coincide with the presence of this species in the area (Fig 2). During this time period, overall coho abundance is of similar magnitude to Chinook abundance, but coho are generally increasing during this time while Chinook are decreasing. Our results suggest that coho salmon may be a more important component of the southern resident whales’ diet than has been previously appreciated. A similar fish-eating population of killer whales in Prince William Sound has been observed to eat primarily coho salmon [35], and our finding of substantial seasonal coho consumption suggests some additional diet similarities between these geographically distinct populations.

Overall, our results clearly support and extend previous estimates of southern resident killer whale diet based on prey remains and non-quantitative (presence/absence) analysis of fecal DNA. However, quantification of DNA sequence abundance in feces has some potential biases and limitations, only some of which are under experimental control, and these limitations should be considered when interpreting our results. First, our study was designed to detect fish prey only; potential prey from other taxa such as marine mammals or invertebrates would not be detected. Although prior studies [9, 10, 17] have indicated that these killer whales specialize on fish prey, this limitation must be kept in mind.

Second, even considering only fish prey, estimation of diet by fecal DNA sequence quantification makes a number of assumptions, including unbiased collection of feces with respect to prey consumed, equal concentration of mitochondria and digestibility in all potential prey species, and non-biased amplification and sequencing of DNA from all consumed prey [25, 36]. Our primers were designed to amplify a large variety of known and potential fish prey species in an unbiased way. The reasonably close correspondence between the expected and observed species compositions in the control pools supports the idea that our results are unlikely to be substantially biased by PCR or sequencing errors, although the higher amplification rates of Chinook and Coho salmon suggest that rare species may be detected less often than they are actually represented in the diet. The controls were performed using extracted DNAs, however, not with equivalent weights or volumes of fish tissue. We have thus not controlled or tested for factors such as differences in mtDNA concentration or digestibility which have been shown to vary between species [25].

Conducting controlled diet experiments in a captive setting would be useful in testing for species quantification biases [36, 37]. At this point we therefore consider the diet estimates generated in this study to be approximations, rather than precise estimates. On the other hand, we are reasonably confident in the absence from the diet of any species in our baseline that were not detected in the millions of sequences that we generated and that were demonstrated to amplify with our primers. Overall, the close correspondence between the fecal DNA results and earlier prey remains results lends credence to the estimates derived from both methods.

Estimating diet by quantifying the relative abundance of prey DNA in a predator’s feces has been applied to a variety of taxa [25, 38], but has had limited applications to cetaceans [26, 39]. This method has the potential to be particularly useful for species, such as cetaceans, that are difficult to observe extensively in the wild. Estimation of predator diets has become increasingly important as formerly rare predator species have increased in abundance due to successful protection under laws such as the U.S. Endangered Species Act or Marine Mammal Protection Act and are preying on other species of concern [6]. Unlike other North Pacific killer whales [40], the southern resident population has not experienced consistent population growth following the implementation of protections in the mid-1970s [14]. An accurate understanding of the whales’ diet, along with the diets of their potential competitors, across the full range of seasons and habitats used by the whales will be helpful in identifying recovery actions that are likely to positively impact this endangered population.

## Supporting Information

S1 Table Table of sampling dates and associated sample groups for the 175 fecal samples used in the analysis. (CSV)

S2 Table Alignment of the primer regions. (DOCX)

S3 Table Distance matrix of number of nucleotide differences among species in the baseline. (CSV)

S4 Table BLAST results of Pacific salmon 16s sequences from GenBank against our baseline. (CSV)

S1 Text Description of methods for estimated daily salmon abundance. (DOCX)
