# Supplementary material for: Estimation of a Killer Whale (Orcinus orca) Population’s Diet Using Sequencing Analysis of DNA from Feces
Source: PLoS One. 2016 Jan 6;11(1):e0144956. doi: 10.1371/journal.pone.0144956 (PMC4703337; doi:10.1371/journal.pone.0144956)
Supplement: S2 Table — (DOCX) [file pone.0144956.s002.docx]

S2 Table

Forward primer alignment (red, underlined) with flanking sequence

|  | **CONSENSUS** |  | CCGTGCGAAGGTAGCGYAATCACTTGTCTTTTAAATGAAGACCTGTATGAATGG |
| --- | --- | --- | --- |
| AF392054 | Salmo salar | Atlantic salmon | ------------------------------------------------------ |
| NC002980 | Oncorhynchus tshawytscha | Chinook salmon | ------------------------------------------------------ |
| NC009263 | Oncorhynchus kisutch | Coho salmon | ------------------------------------------------------ |
| NC009261 | Oncorhynchus keta | Chum salmon | ------------------------------------------------------ |
| AF312573 | Oncorhynchus mykiss | Steelhead | ------------------------------------------------------ |
| EF458341 | Clupea pallasii | herring | ------A---------------A------------------------------- |
| EF458415 | Hypomesus pretiosus | surf smelt | ----------------------------C------------------------- |
| EF119256 | Cymatogaster aggregata | shiner perch | ------A----------------------------------------------- |
| EF119283 | Rhacochilus vacca | pile surfperch | ------A----------------------------------------------- |
| EF446517 | Sebastes saxicola | stripetail rockfish | ------A----------------------------------------------- |
| DQ678228 | Sebastes elongatus | greenstriped rockfish | ------A----------------------------------------------- |
| EF446598 | Sebastes pinniger | canary rockfish | ------A----------------------------------------------- |
| EF446522 | Sebastes ruberrimus | yelloweye rockfish | ------A----------------------------------------------- |
| EF446592 | Sebastes proriger | redstripe rockfish | ------A----------------------------------------------- |
| EF446569 | Sebastes dallii | calico rockfish | ------A----------------------------------------------- |
| EF446567 | Sebastes serriceps | treefish | ------A----------------------------------------------- |
| DQ678218 | Sebastes carnatus | gopher rockfish | ------A----------------------------------------------- |
| DQ678217 | Sebastes atrovirens | kelp rockfish | ------A----------------------------------------------- |
| DQ678223 | Sebastes nebulosus | china rockfish | ------A----------------------------------------------- |
| DQ678307 | Sebastes auriculatus | brown rockfish | ------A----------------------------------------------- |
| EF446582 | Sebastes rastrelliger | grass rockfish | ------A----------------------------------------------- |
| DQ678220 | Sebastes chrysomelas | Black-and-yellow rockfish | ------A----------------------------------------------- |
| EF446562 | Sebastes caurinus | copper rockfish | ------A----------------------------------------------- |
| EF446599 | Sebastes maliger | quillback rockfish | ------A----------------------------------------------- |
| DQ678239 | Sebastes flavidus | yellowtail rockfish | ------A----------------------------------------------- |
| EF446596 | Sebastes melanops | black rockfish | ------A----------------------------------------------- |
| EF446580 | Sebastes nigrocinctus | tiger rockfish | ------A----------------------------------------------- |
| DQ678235 | Sebastes entomelas | widow rockfish | ------A----------------------------------------------- |
| DQ678252 | Sebastes mystinus | blue rockfish | ------A----------------------------------------------- |
| EF446565 | Sebastes paucispinus | boccacio | ------A----------------------------------------------- |
| EF458482 | Anaoplopoma fimbria | sablefish | ------------------------------------------------------ |
| EF458426 | Ophiodon elongatus | lingcod | -------------------------------------------C---------- |
| EF119280 | Hydrolagus colliei | spotted ratfish | ----------------T------------------------------------- |
| EF119335 | Squalus acanthias | spiny dogfish | ----------------T------------------------------------- |
| EF458337 | Merluccius productus | Pacific Hake | ----------------T------------------------------------- |
| EF119325 | Gadus chalcogrammus | walleye pollock | ----------------T------------------------------------- |
| EF458431 | Microgadus proximus | pacific tomcod | ----------------T------------------------------------- |
| EF119278 | Raja rhina | longnose skate | ------A---------T--------------------------C---------- |
| EF119303 | Microstomus pacificus | Dover sole | ------A---------T------------------------------------- |
| FJ870421 | Hippoglossus stenolepis | Pacific halibut | ------A---------T------------------------------------- |
| EF119312 | Psettichthys melanostictus | pacific sand sole | ------A---------T------------------------------------- |
| EF119293 | Lepidopsetta bilineata | rock sole | ------A---------T------------------------------------- |
| EF119308 | Platichthys stellatus | starry flounder | ------A---------T------------------------------------- |
| EF458362 | Lepidopsetta polyxystra | norther rock sole | ------A---------T------------------------------------- |
| EF119289 | Parophrys vetulus | english sole | ------A---------T------------------------------------- |
| AY835654 | Scorpaenichthys marmoratus | cabezon | -------------------------------------G-----C---------- |
| EF458461 | Myoxocephalus polyacanthocephalus | great sculpin | -------------------------------------G-----C---------- |
| EF458404 | Trichodon trichodon | pacific sandfish | -------------------------------------G-----C---------- |
| EU685093 | Orcinus orca | Killer whale | ---------------AT----------TC-C-----A-G---T----------- |
| JF504729 | Phocoednoides dalli | Dall's porpoise | ---------------AT----------TC-C-----A-G---T----------- |
| AB481401 | Phocoena phocoena | harbor porpoise | ---------------AT----------TC-C-----A-G---T----------- |
| AM181032 | Phoca vitulina | harbor seal | ---------------AT-----T----TC-A-----A-G---T--------C-- |
|  | **CONSENSUS** |  | CCGTGCGAAGGTAGCGCAATCACTTGTCTTTTAAATGAAGACCTGTATGAATGG |

Reverse Primer Alignment

|  | **CONSENSUS** |  | ACCCTAGGGATAACAGCGCAATCCCCTTTTAGA | |
| --- | --- | --- | --- | --- |
| AF392054 | Salmo salar | Atlantic salmon | ------------------------T--CCC--- | |
| NC002980 | Oncorhynchus tshawytscha | Chinook salmon | ------------------------T--CCC--- | |
| NC009263 | Oncorhynchus kisutch | Coho salmon | ------------------------T--CCC--- | |
| NC009261 | Oncorhynchus keta | Chum salmon | ------------------------T--CCC--- | |
| AF312573 | Oncorhynchus mykiss | Steelhead | ------------------------T--CCC--- | |
| EF458341 | Clupea pallasii | herring | -------------------------T-CCC--- | |
| EF458415 | Hypomesus pretiosus | surf smelt | ------------------------T--CCC--- | |
| EF119256 | Cymatogaster aggregata | shiner perch | -------------------------T-C----- | |
| EF119283 | Rhacochilus vacca | pile surfperch | -----------------------T---C----- | |
| EF446517 | Sebastes saxicola | stripetail rockfish | --------------------------------- | |
| DQ678228 | Sebastes elongatus | greenstriped rockfish | --------------------------------- |  |
| EF446598 | Sebastes pinniger | canary rockfish | --------------------------------- | |
| EF446522 | Sebastes ruberrimus | yelloweye rockfish | --------------------------------- | |
| EF446592 | Sebastes proriger | redstripe rockfish | --------------------------------- | |
| EF446569 | Sebastes dallii | calico rockfish | --------------------------------- | |
| EF446567 | Sebastes serriceps | treefish | --------------------------------- | |
| DQ678218 | Sebastes carnatus | gopher rockfish | --------------------------------- | |
| DQ678217 | Sebastes atrovirens | kelp rockfish | --------------------------------- | |
| DQ678223 | Sebastes nebulosus | china rockfish | --------------------------------- | |
| DQ678307 | Sebastes auriculatus | brown rockfish | --------------------------------- | |
| EF446582 | Sebastes rastrelliger | grass rockfish | --------------------------------- | |
| DQ678220 | Sebastes chrysomelas | Black-and-yellow rockfish | --------------------------------- | |
| EF446562 | Sebastes caurinus | copper rockfish | --------------------------------- | |
| EF446599 | Sebastes maliger | quillback rockfish | --------------------------------- | |
| DQ678239 | Sebastes flavidus | yellowtail rockfish | --------------------------------- | |
| EF446596 | Sebastes melanops | black rockfish | --------------------------------- | |
| EF446580 | Sebastes nigrocinctus | tiger rockfish | --------------------------------- | |
| DQ678235 | Sebastes entomelas | widow rockfish | --------------------------------- | |
| DQ678252 | Sebastes mystinus | blue rockfish | --------------------------------- | |
| EF446565 | Sebastes paucispinus | boccacio | --------------------------------- | |
| EF458482 | Anaoplopoma fimbria | sablefish | ------------------------T-------- | |
| EF458426 | Ophiodon elongatus | lingcod | ------------------------T-------- | |
| EF119280 | Hydrolagus colliei | spotted ratfish | ------------------------TT—-CC--- | |
| EF119335 | Squalus acanthias | spiny dogfish | ------------------------TT-CCC--- | |
| EF458337 | Merluccius productus | Pacific Hake | ----C----------------------C-C--- | |
| EF119325 | Gadus chalcogrammus | walleye pollock | ---------------------------C-C--- | |
| EF458431 | Microgadus proximus | pacific tomcod | ---------------------------C-C--- | |
| EF119278 | Raja rhina | longnose skate | --------------------------------- | |
| EF119303 | Microstomus pacificus | Dover sole | --------------------------------- | |
| FJ870421 | Hippoglossus stenolepis | Pacific halibut | --------------------------------- | |
| EF119312 | Psettichthys melanostictus | pacific sand sole | --------------------------------- | |
| EF119293 | Lepidopsetta bilineata | rock sole | --------------------------------- | |
| EF119308 | Platichthys stellatus | starry flounder | --------------------------------- | |
| EF458362 | Lepidopsetta polyxystra | norther rock sole | --------------------------------- | |
| EF119289 | Parophrys vetulus | english sole | --------------------------------- | |
| AY835654 | Scorpaenichthys marmoratus | cabezon | ------------------------T-------- | |
| EF458461 | Myoxocephalus polyacanthocephalus | great sculpin | ------------------------T-------- | |
| EF458404 | Trichodon trichodon | pacific sandfish | ------------------------T-------- | |
| EU685093 | Orcinus orca | Killer whale | ------------------------TA--C---- | |
| JF504729 | Phocoednoides dalli | Dall's porpoise | ------------------------TA--C---- | |
| AB481401 | Phocoena phocoena | harbor porpoise | ------------------------TA--C---- | |
| AM181032 | Phoca vitulina | harbor seal | ------------------------TG---G--- | |
|  | **CONSENSUS** |  | ACCCTAGGGATAACAGCGCAATCCCCTTTTAGA | |

qPCR reverse primer alignment

|  | **CONSENSUS** |  | ACATAAGACGAGAAGACCCTATGGAGCTTT |
| --- | --- | --- | --- |
| AF392054 | Salmo salar | Atlantic salmon | ------------------------------ |
| NC002980 | Oncorhynchus tshawytscha | Chinook salmon | ------------------------------ |
| NC009263 | Oncorhynchus kisutch | Coho salmon | ------------------------------ |
| NC009261 | Oncorhynchus keta | Chum salmon | ------------------------------ |
| AF312573 | Oncorhynchus mykiss | Steelhead | ------------------------------ |
| EF458341 | Clupea pallasii | herring | -T-C-------------------------- |
| EF458415 | Hypomesus pretiosus | surf smelt | G----------------------------- |
| EF119256 | Cymatogaster aggregata | shiner perch | ------------------------------ |
| EF119283 | Rhacochilus vacca | pile surfperch | ----T------------------------- |
| EF446517 | Sebastes saxicola | stripetail rockfish | G----------------------------- |
| DQ678228 | Sebastes elongatus | greenstriped rockfish | ------------------------------ |
| EF446598 | Sebastes pinniger | canary rockfish | ------------------------------ |
| EF446522 | Sebastes ruberrimus | yelloweye rockfish | ------------------------------ |
| EF446592 | Sebastes proriger | redstripe rockfish | ------------------------------ |
| EF446569 | Sebastes dallii | calico rockfish | ------------------------------ |
| EF446567 | Sebastes serriceps | treefish | ------------------------------ |
| DQ678218 | Sebastes carnatus | gopher rockfish | ------------------------------ |
| DQ678217 | Sebastes atrovirens | kelp rockfish | ------------------------------ |
| DQ678223 | Sebastes nebulosus | china rockfish | ------------------------------ |
| DQ678307 | Sebastes auriculatus | brown rockfish | ------------------------------ |
| EF446582 | Sebastes rastrelliger | grass rockfish | ------------------------------ |
| DQ678220 | Sebastes chrysomelas | Black-and-yellow rockfish | ------------------------------ |
| EF446562 | Sebastes caurinus | copper rockfish | ------------------------------ |
| EF446599 | Sebastes maliger | quillback rockfish | ------------------------------ |
| DQ678239 | Sebastes flavidus | yellowtail rockfish | ------------------------------ |
| EF446596 | Sebastes melanops | black rockfish | ------------------------------ |
| EF446580 | Sebastes nigrocinctus | tiger rockfish | ------------------------------ |
| DQ678235 | Sebastes entomelas | widow rockfish | ------------------------------ |
| DQ678252 | Sebastes mystinus | blue rockfish | ------------------------------ |
| EF446565 | Sebastes paucispinus | boccacio | ------------------------------ |
| EF458482 | Anaoplopoma fimbria | sablefish | ------------------------------ |
| EF458426 | Ophiodon elongatus | lingcod | ------------------------------ |
| EF119280 | Hydrolagus colliei | spotted ratfish | ------------------------------ |
| EF119335 | Squalus acanthias | spiny dogfish | C---T------------------------C |
| EF458337 | Merluccius productus | Pacific Hake | -T------------------G--------- |
| EF119325 | Gadus chalcogrammus | walleye pollock | ------------------------------ |
| EF458431 | Microgadus proximus | pacific tomcod | ------------------------------ |
| EF119278 | Raja rhina | longnose skate | ------------------------------ |
| EF119303 | Microstomus pacificus | Dover sole | ------------------------------ |
| FJ870421 | Hippoglossus stenolepis | Pacific halibut | C----------------------------- |
| EF119312 | Psettichthys melanostictus | pacific sand sole | C----------------------------- |
| EF119293 | Lepidopsetta bilineata | rock sole | C----------------------------- |
| EF119308 | Platichthys stellatus | starry flounder | C----------------------------- |
| EF458362 | Lepidopsetta polyxystra | norther rock sole | C----------------------------- |
| EF119289 | Parophrys vetulus | english sole | C----------------------------- |
| AY835654 | Scorpaenichthys marmoratus | cabezon | ------------------------------ |
| EF458461 | Myoxocephalus polyacanthocephalus | great sculpin | ------------------------------ |
| EF458404 | Trichodon trichodon | pacific sandfish | ------------------------------ |
| EU685093 | Orcinus orca | Killer whale | C----------------------------- |
| JF504729 | Phocoednoides dalli | Dall's porpoise | ------------------------------ |
| AB481401 | Phocoena phocoena | harbor porpoise | ------------------------------ |
| AM181032 | Phoca vitulina | harbor seal | T----------------------------- |
|  | **CONSENSUS** |  | ACATAAGACGAGAAGACCCTATGGAGCTTT |
