# Supplementary material for: Estimation of a Killer Whale (Orcinus orca) Population’s Diet Using Sequencing Analysis of DNA from Feces
Source: PLoS One. 2016 Jan 6;11(1):e0144956. doi: 10.1371/journal.pone.0144956 (PMC4703337; doi:10.1371/journal.pone.0144956)
Supplement: S1 Text — (DOCX) [file pone.0144956.s005.docx]

S1 Text - Supplemental methods

Estimation of daily salmon abundance

We approximated the daily abundance of Chinook, sockeye and coho salmon available to the whales in the San Juan Islands area. The purpose of these approximations was to provide a rough indicator of the relative abundance of these three species available to the whales during the time period of our sampling. The basic approach for each species was to use catch-per-unit-effort (CPUE) data from state-sponsored test fisheries, scaled by an estimate of the total annual abundance of the runs to obtain estimated daily abundance. A smoothed curve was then fit to these daily abundance estimates to reduce noise from environmental and sampling variance. Details for each species are described below.

Chinook salmon

A prior study found that the Chinook salmon consumed by the southern resident killer whales in the San Juan Islands area originate primarily from the Fraser River, British Columbia [1]. The Canadian Department of Fisheries and Oceans conducts an annual test fishery in the Lower Fraser River, which typically operates daily from April 1 to August 31, and every other day from September into mid-October when it alternates daily with a test fishery targeting chum salmon (http://www.pac.dfo-mpo.gc.ca/fm-gp/fraser/docs/commercial/albionchinook-quinnat-eng.html). The daily CPUE data for the years of our study were downloaded from the CDFO web site (link above). Based an assumed 10 day travel time from the west side of San Juan Island to the mouth of the Fraser River [2], we subtracted 10 days from the test fisheries dates to obtain a rough daily index of Chinook abundance available to the whales. In order to convert this index into an approximate estimate of actual abundance, we multiplied the daily CPUE data (rescaled to sum to 1) by the annual estimates of Chinook salmon returning to the Fraser River. These total run size estimates were obtained from summing the escapement estimates for the Fraser River Chinook runs summarized by the Pacific Salmon Commission’s Chinook Technical Committee in Appendix B.5 of their Annual Report of Catch and Escapement [3]. Finally, to reduce the effects of environmental variation on the CPUE data, we used local polynomial regression (loess function in the R statistical package [4]) to fit a smoothed curve to the daily estimated abundances.

Coho salmon

Smoothed daily estimates of coho salmon abundance were obtained in the same manner as described for Chinook, with the following exceptions. Coho salmon are incidentally caught in the same Fraser River test fishery described above for Chinook salmon. We used the coho CPUE data from this fishery combined with those from the chum test fishery operated by the same vessel (<http://www-ops2.pac.dfo-mpo.gc.ca/fos2_Internet/Testfish/rptcsbdparm.cfm?stat=CPTFM&fsub_id=227>) as an estimate of coho run timing to the Fraser River, also subtracting 10 days to account for fish travel time. We did not have any information on which coho stocks the whales are consuming, so we used the summed escapement estimates of coho salmon to the Fraser, Georgia Strait, and northern Puget Sound (Skagit, Snohomish, and Stillaguamish Rivers) as an estimate of total coho abundance in the area. Escapement estimates for 2006-2008 were obtained from the 2013 Pacific Salmon Commission’s Coho Technical Committee [5], and those from 2010 and 2011 were obtained from unpublished Coho Technical Committee annual reports (Laurie Weitkamp (NMFS, CTC), personal communication).

Sockeye salmon

Smoothed daily estimates of sockeye salmon abundance were obtained in the same manner as described for Chinook, with the following exceptions. A daily abundance index of sockeye salmon in Haro Strait was derived from the estimated daily Area 20 test fishery CPUE, plus 5 days to account for average travel time for migrating sockeye traveling between Area 20 and mid-Haro Strait on the west side of San Juan. The Fraser River sockeye abundance estimate for Haro Strait was calculated as the total Area 20 run size, less catches in Areas 20, 7 and 7A. Run size and catch data for Fraser sockeye salmon were obtained from the Pacific Salmon Commission (personal communication from C. Michielsens and M. Lapointe to Tim Tynan (NMFS) March 2014).

1. Hanson M, Baird R, Ford J, Hempelmann-Halos J, Van Doornik D, Candy J, et al. Species and stock identification of prey consumed by endangered southern resident killer whales in their summer range. Endangered Species Research. 2010;11(1):69-82. doi: 10.3354/esr00263.

2. Ayres KL, Booth RK, Hempelmann JA, Koski KL, Emmons CK, Baird RW, et al. Distinguishing the Impacts of Inadequate Prey and Vessel Traffic on an Endangered Killer Whale *Orcinus orca* Population. PLoS ONE. 2012;7(6):e36842.

3. Pacific Salmon Commission. Joint Chinook Technical Committee Report Annual Report Of Catch And Escapement For 2012 Report Tcchinook (13)-01. Available at [http://www.psc.org/publications_tech_techcommitteereport.htm - TCCHINOOK](http://www.psc.org/publications_tech_techcommitteereport.htm#TCCHINOOK). 2013.

4. R Core Team. R: A language and environment for statistical computing. R Foundation for Statistical Computer, Vienna, Austria. <http://www.R-project.org>. 2012.

5. Pacific Salmon Commission. Pacific Salmon Commission Joint Coho Technical Committee 1986-2009 Periodic Report. Revised. Report Tccoho (13)–1. Available at: [http://www.psc.org/publications_tech_techcommitteereport.htm - TCCOHO](http://www.psc.org/publications_tech_techcommitteereport.htm#TCCOHO). 2013.
